# Decreasing leakage during continuous femoral nerve catheter fixation using 2-octyl cyanoacrylate glue (Dermabond®): a randomized controlled trial

**DOI:** 10.1186/s12871-021-01386-7

**Published:** 2021-06-11

**Authors:** Theerawat Chalacheewa, Vanlapa Arnuntasupakul, Lisa Sangkum, Rungrawan Buachai, Jiravud Chanvitayapongs

**Affiliations:** 270 Department of Anesthesiology, Faculty of Medicine, Ramathibodi hospital, Mahidol University, Rama VI road, Phayathai, Ratchatewi, Bangkok, 10400 Thailand

**Keywords:** Continuous peripheral nerve catheters, Pericatheter leakage, Catheter displacement, Dermabond

## Abstract

**Background:**

Continuous peripheral nerve catheters (CPNCs) have been used for postoperative pain relief. A common problem encountered with CPNCs is pericatheter leakage, which can lead to dressing adhesive failure. Frequent dressing changes increase the risk of catheter dislodgement and infections. Adhesive glue is effective in securing the peripheral nerve catheter and decreasing leakage around the catheter insertion site. This study aimed to evaluate the incidence of pericatheter leakage with fixation using 2-octyl cyanoacrylate glue (Dermabond**®**) as compared to sterile strips.

**Methods:**

Thirty patients undergoing unilateral total knee arthroplasty (TKA) with continuous femoral nerve catheter for postoperative analgesia were randomized into the catheter fixation with 2-octyl cyanoacrylate glue (Dermabond**®**) group or the sterile strip group. The primary outcome was the incidence of pericatheter leakage. Secondary outcomes included the frequent of catheter displacement, the difficulty of catheter removal, pain score and patient satisfaction.

**Results:**

The incidence of pericatheter leakage at 24 and 48 h was 0% versus 93 and 0% versus 100% in the Dermabond**®** and sterile strip groups, respectively (*P* < 0.001). The incidence of displacement at 24 and 48 h was 6.7% versus 93.3 and 6.7% versus 100% in the Dermabond® and sterile strip, respectively (*P* < 0.001). There was no difference in numeric rating scale, difficulty of catheter removal, or satisfaction scores between groups.

**Conclusions:**

Catheter fixation with 2-octyl cyanoacrylate glue (Dermabond**®**) decreased the incidence of pericatheter leakage, as well as catheter displacement, over 48 h as compared to sterile strip fixation.

**Trial registration:**

This trial was registered on Thai clinical trial registry: TCTR20200228002, registered 24 February 2020- Retrospectively registered.

## Background

Continuous peripheral nerve catheters (CPNCs) provide sustained postoperative pain relief with an opioid-sparing effect, improved rehabilitation, and patient satisfaction [1–3]. The challenge to maintain CPNC function relates to securing the catheter in the correct position, especially in the freely mobile sites of the neck and limbs. Moreover, by preventing pericatheter leakage, the risk of dislodgement and infection can be deceased [4]. Several methods with which to secure catheters have been studied, such as suturing, cutaneous sutures [5], and retrograde subcutaneous tunneling [6]. However, accidental dislodgment and pericatheter leakage are still frequent problems.

In our institute, patients undergoing TKA received continuous femoral nerve blocks with stimulating catheters for postoperative analgesia. We used stimulating catheters to allow confirm that the catheter tip was located near the target nerve. Because stimulating catheters are placed using the catheter-through-a-needle method, this causes the needle puncture hole diameter to become larger than the catheter. Additionally, we used sterile strips for the fixation of catheters for which we found a high incidence of perivascular leakage. Thus, we attempted to look for the method to prevent such incidents. Previous studies [7–10] have shown that adhesive glue was an effective method of peripheral nerve catheter fixation; however, leakage around the pericatheter was only the secondary outcome of these studies. The fixation of the peripheral catheter with 2-ocylcyanoacrylate (Dermabond®) may improve leakage by sealing effect at the puncture site, and securing the continuous femoral nerve catheter with 2-ocylcyanoacrylate (Dermabond®) would reduce the incidence of pericatheter leakage within the first 48 h. The primary aim of this study is to evaluate the incidence of pericatheter leakage by using sterile stripe or 2-ocylcyanoacrylate (Dermabond®) in continuous femoral catheter. The secondary outcomes were catheter displacement, numeric rating scale score (NRS), difficulty of catheter removal, and patient satisfaction with analgesia.

## Methods

### Study participants

After receiving approval by the Ramathibodi hospital research ethics board (ID 06–61-10, Date of approval: February 23, 2018). This trial was registered in Thai clinical trial registry (TCTR20200228002, Date of registration: February 24, 2020). A late registration did not experience any changes in the study protocol. The reporting of this study was performed by adhering to the Consolidated Standards of Reporting Trials (CONSORT) statement for the reporting of randomized trial. Written informed consent was obtained from all participants. We enrolled 30 adult patients aged 40–80 years who had American Society of Anesthesiologists physical status IIV and were scheduled to undergo total knee arthroplasty (TKA). We excluded patients who refused to participate in the study, as well as patients with contraindications for performing femoral nerve blocks and spinal anesthesia (e.g., localized infection at puncture site, history of the femoral nerve neuropathy, and history of local anesthetic drug/adhesive glue allergy).

### Randomization

Our research coordinator, who was not involved in the study, performed a computer-generated simple 1:1 ratio to assign patients into to 2-octyl cyanoacrylate glue (Dermabond®) (Dermabond group) or normal practice fixation groups (control group). The data on the allocation of patients to each group were concealed in a sealed envelope. The anesthesiologist who performed the femoral nerve block opened the envelope just prior to block performance.

### Femoral nerve block

**Fig. 1:**
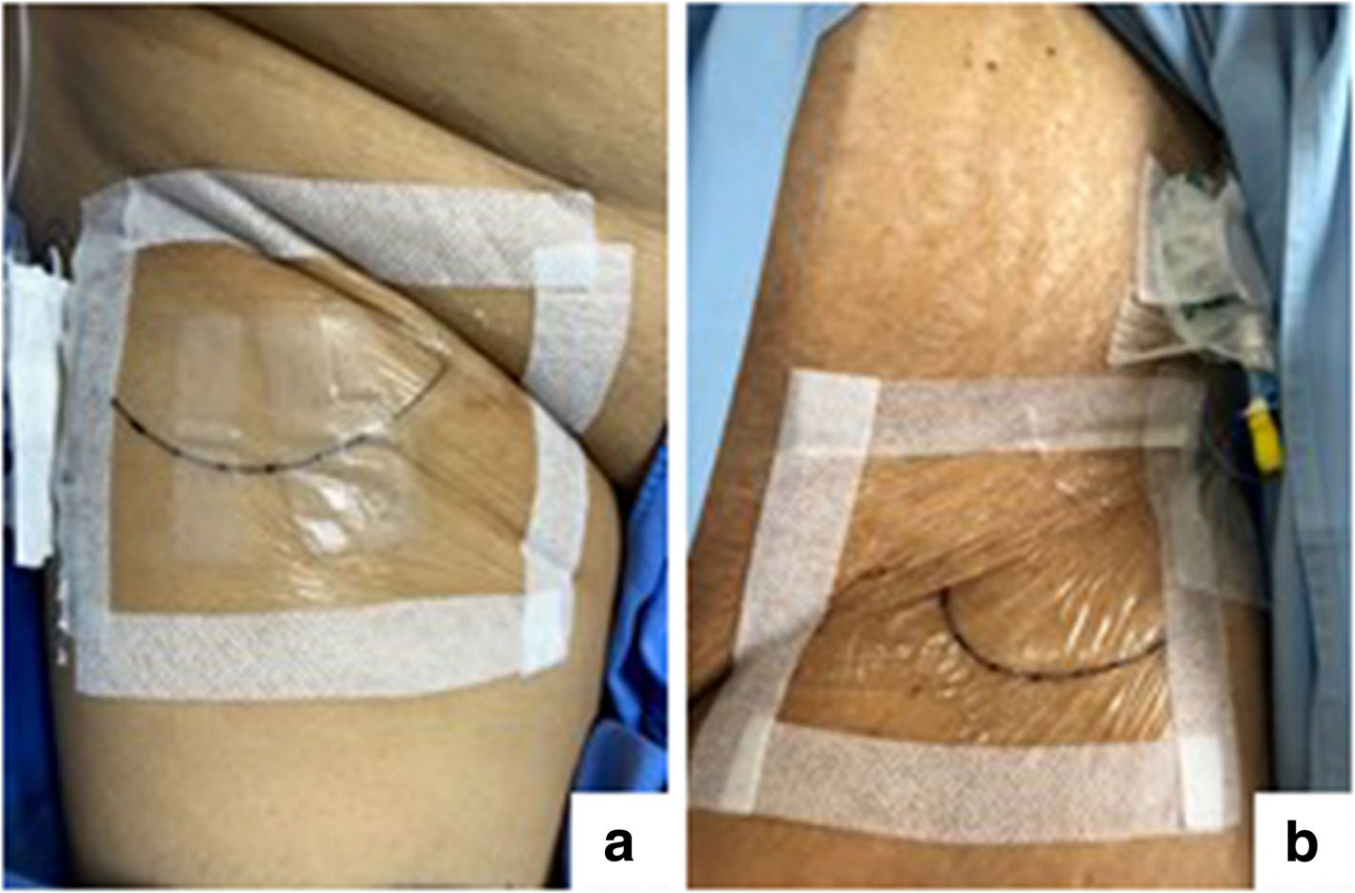
**a** Catheter is secured with sterile strip at right groin. **b** Catheter is secured with 2-octyl cyanoacrylate liquid adhesive (Dermabond®) at left groin

For patients undergoing unilateral TKA, femoral nerve catheters were inserted pre-operatively using a nerve stimulator. A Stimulong® 18-gauge needle was inserted at 1.5 cm lateral to the femoral artery. The nerve stimulator was initially set at 2 Hz and 1.5 mA. When quadriceps contraction was detected, the current was decreased, and the needle position was optimized for contraction at a current output of 0.2–0.5 mA. The Stimulong® 20-gauge catheter was attached to the nerve stimulator, and the catheter was slowly threaded until a depth of 3–5 cm from the needle tip was reached, while maintaining quadriceps contraction at a current ≤0.5 mA. The needle was withdrawn, and the patients were divided into two groups: the Dermabond group and the control group. In the Dermabond group, the catheter was sealed with 2-octyl cyanoacrylate liquid adhesive (Dermabond®), and the area was covered with a transparent dressing (Tegaderm™). In the control group, the catheter was secured with a sterile strip, and the area was covered with transparent dressing (Tegaderm™) (Fig. 1).

Intra-operatively, spinal anesthesia was performed with a 27-gauge quincke needle using isobaric bupivacaine at 10–15 mg from the second to the fourth lumbar levels. No local anesthetics were given through the femoral catheter during surgery. In the recovery room, the femoral catheter received a bolus containing 15 ml of bupivacaine 0.125%, and then, continuous infusion with bupivacaine 0.08% at 5 mL/h began. Every patient received acetaminophen at 1000 mg orally every 6 h and naproxen at 250 mg orally for every 8 h for 3 days. If the postoperative pain score was greater than 4 out of 10, IV morphine at 3 mg was administered as rescue analgesia.

### Postoperative follow-up

The incidence of pericatheter leakage, catheter displacement, difficult catheter removal, pain score, and patient satisfaction were assessed via the acute pain service nurse.

Pericatheter leakage was classified as 1) no leakage or 2) leakage: fluid was seen under the transparent dressing (Tegaderm™).

The catheter displacement was defined as catheter migration from the initial recorded depth of insertion. Catheter dislodgement was defined as complete catheter removal from the skin. Catheter removal was reported as “easy” or “difficult.” Post-operative pain was assessed in the postanesthetic care unit (PACU) and, then, at 24 h and 48 h using a 10-point numerical rating scale. Patient satisfaction with analgesia was recorded using a 0–10 scale, with 0 = very dissatisfied and 10 = completely satisfied (excellent).

### Statistical methodology

The demographic characteristics of the subjects in each randomized controlled study were analyzed. Continuous variables are reported using mean and standard deviation values or median and range values. Categorical variables are presented using counts and percentages and tested using Chi-Square or Fisher’s exact test, as appropriate. Continuous variables were tested for normality with the Shapiro-Wilk test. A student’s t-test or Mann-Whitney test was used for group comparisons as appropriate. A *p*-value < 0.05 implied statistical significance. The statistical software SPSS 20.0 for Windows was used for data analyses.

Based on institutional pilot data from acute pain service found 50% leakage when we used sterile strip. We expected that dermabond could reduce the leakage to zero. There is at least 80% power to reveal a clinically, with a two-sided type-I error rate of 0.05. A sample size of eleven subjects in each arm of each group was considered adequate. An additional four subjects per group were recruited to prevent the loss of power because of early withdrawal or protocol violations. Thus, 15 subjects per group was the derived sample size.

## Results

**Fig. 2:**
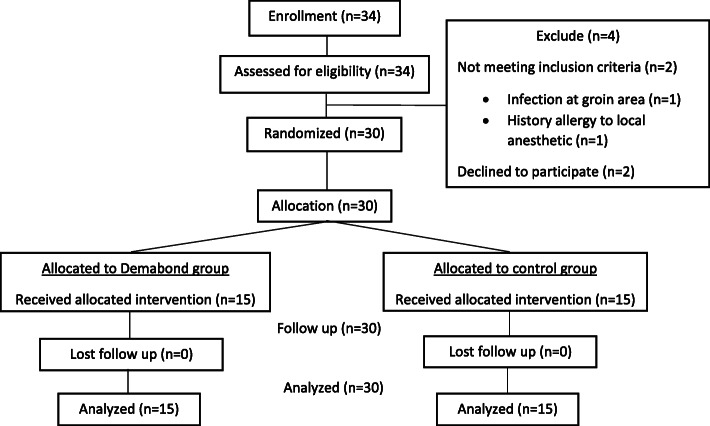
CONSORT diagram showing the flow of the study participants

The distribution and allocation of the patients are outlined in the CONSORT flow diagram (Fig. 2).

In both groups, there was no significant difference in the sex, age, body mass index (BMI), and ASA physical status of the subjects (Table 1).

**Table 1:** Patients’ demographic data

	Dermabond group (15)	Control group (15)	*P*-value
Male/Female, n	0/15	3/12	0.224
Age (y), mean ± SD	68.2 ± 7.5	66.33 ± 6.77	0.48
BMI (kg.m^−2^), mean ± SD	25.99 ± 3.67	27.41 ± 3.26	0.272
ASA I/II/III, n	0/8/7	1/8/6	> 0.999

*ASA* American society of anesthesiologist, *BMI* Body mass index, SD: Standard deviation

The incidence of pericatheter leakage at 24 and 48 h was significant lower in the Dermabond group as compared to the control group (at 24 h: 0% versus 93.33%, *P* < 0.001, 48 h: 0% versus 100%, *P* < 0.001). The incidence of catheter displacement was significantly lower in the Dermabond group as compared to the control group (at 24 h: 6.7% versus 73.3%, *P* < 0.001, 48 h: 6.7% versus 93.3%, *P* < 0.001), as well as the incidence of CPNC dressing changes. There was no difference in the numeric rating scale at 24 and 48 h between groups (24-h NRS: 2 (0–7) versus 2 (0–8), *P* = 0.54), 48-h NRS: 2 (0–7) versus 2 (0–7), *P* = 0.81) or any difference in satisfaction score (satisfaction score: Dermabond vs. control group: 9.13 ± 1.13 vs 9.07 ± 1.28, *P* = 0.881) (Table 2). In both groups, there was no incidence of dislodgement or catheter removal difficulty.

**Table 2:** Catheter- and subject-related outcomes for the Dermabond as compared with the control groups

	Dermabond group	Control group	*P*-value
**Leakage, n (%)**			
**24 h after operation**	**0 (0%)**	**14 (93.33%)**	< 0.001*
**48 h after operation**	**0 (0%)**	**15 (100%)**	< 0.001*
**Displacement, n** (%)			
24 h after operation	1 (6.67%)	11 (73.33%)	< 0.001*
48 h after operation	1 (6.67%)	14 (93.33%)	< 0.001*
**Change dressing, n (%)**			
24 h after operation	0	1 (0–3)	0.001
48 h after operation	0	1 (0–4)	< 0.001
**NRS**			
24 h after operation	2 (0–7)	2 (0–8)	0.539
48 h after operation	2 (0–7)	2 (0–7)	0.806
**Satisfaction score, mean ± SD**	9.13 ± 1.13	9.07 ± 1.28	0.881

*NRS* Numeric rating scale, *SD* Standard deviation

## Discussion

This prospective randomized trial study revealed a significant improvement of pericatheter leakage with 2-octylcyanoacrylate (Dermabond®) fixation as compared with sterile strips. It also prevents the occurrence of catheter displacement and frequent dressing changes while maintaining high-quality pain control and satisfaction scores.

In brief, 2-octylcyanoacrylate (Dermabond®) is a liquid monomer that spontaneously polymerizes in the presence of moisture to create a waterproof bond with the surrounding epidermis. Several studies have compared this surgical adhesive to sutures for use in the closure of lacerations in both trauma and elective surgery. Therefore, many studies have evaluated the efficacy of Dermabond with CPNCs. Our data clearly reveal that 2-octylcyanoacrylate (Dermabond®) improves pericatheter leakage by sealing the puncture site and securing the catheter tightly by the skin.

Rika Nogawa et al. [11] compared the incidence of leakage from the catheter insertion site during continuous femoral nerve block when using the catheter-through-the-needle and catheter-over-needle methods. They found the incidence of pericatheter leakage in eleven of 20 patients in the catheter-through-needle group. However, the pericatheter leakage in Nogawa’s study was less than was observed in our study. We found 14 of 15 patients with catheter leakage at 24 h and 15 of 15 patients at 48 h post-operatively.

The difference in results may have been caused by the different femoral nerve block techniques used. While Nogawa R. et al. used ultrasound, we used a landmark technique that may result in several puncture holes in the patient’s body. Unfortunately, we did not record the number of punctures that occurred. However, the severity of this incidence could be alleviated using Dermabond, and this could be confirmed by a new study. Gurnaney et al. reported the incidence of pericatheter leakage both before and after using Dermabond as a catheter fixation protocol in CPNCs, which helped decrease the leakage rate from 3.87 to 0.56% [10]. The effectiveness of Dermabond was also seen in our study, and it helped in reducing the leakage to 0%. Moreover, Dermabond reduced pericatheter bleeding from several puncture sites. Dermabond did not increase the difficulty of catheter removal at the moment of planned discontinuation. Even though the cost of Dermabond is greater than sterile strips, it could lower the indirect costs of CPNC failure, such as additional nursing care and medications. Given the results, the fixation of CPNCs with Dermabond may be useful in ambulatory settings because the incidences of pericatheter leakage, displacement, and dislodgment are reduced and less patient self-care and inconvenience is required at home.

Even though the registration in Thai Clinical Trial Registry was performed after the study was completed, this study was done in same protocol that sent to Ramathibodi Ethic Committee. Nonetheless, Our study still has some limitations. First, in our study, the assessor and patient were not blinded to the fixation technique, potentially contributing to observer bias in the study. However, our leakage and displacement criteria are objectively evaluated, thereby reducing the potential for observer bias. Second, there are several perineural catheter systems available in the market, and these results may not be generalized to all types of peripheral nerve block catheters, such as those involving the catheter-over-a-needle technique. Because our institute mainly place CPNCs using the catheter-through-a-needle technique, we found fixation using Dermabond to significant reduce the occurrence of pericatheter leakage. Third, the results of our study were obtained from a single center that has vast experience in the catheter care process. This experience must be considered when attempting to replicate the results in different clinical settings. Lastly, our study did not include the incidence of leakage or displacement after 48 h, because our post-operative knee replacement protocol stopped local anesthetic infusion on Postoperative Day 2 to enhance patient muscle strength for ambulation.

## Conclusions

Catheter fixation with 2-octylcyanoacrylate (Dermabond®) reduced the incidence of pericatheter leakage, as well as catheter displacement, over 48 h in a continuous femoral block as compared to sterile strips.

## Data Availability

The datasets analyzed during the current study are available from the corresponding author upon reasonable request.

## Abbreviations

BMI: Body mass index
CPNCs: Continuous peripheral nerve catheters (CPNCs)
NRS: Numeric rating scale score
PACU: Post-anesthetic care unit
TKA: Total knee arthroplasty
